# Brain health: a new concept to the neurologist – an initiative of the Lifestyle Medicine Commission of the Brazilian Academy of Neurology

**DOI:** 10.1055/s-0045-1811234

**Published:** 2025-08-31

**Authors:** Luis Daniel Silva Pilatti, Conrado Regis Borges, Samira Luisa Apóstolos-Pereira, Marcelo Rezende Young Blood, Raphael Machado Castilhos, Brenda Leticia Lopes Batista, Fabiano Moulin de Moraes, Marcos Christiano Lange

**Affiliations:** 1 Universidade Federal do Paraná, Complexo Hospital de Clínicas, Divisão de Neurologia, Curitiba PR, Brazil.; 2 Hospital Sírio-Libanês, Divisão de Neurologia, São Paulo SP, Brazil.; 3 Universidade de São Paulo, Faculdade de Medicina, Departamento de Neurologia, São Paulo SP, Brazil.; 4 Universidade Estadual de Ponta Grossa, Hospital Universitário, Divisão de Neurologia, Ponta Grossa PR, Brazil.; 5 Hospital de Clínicas de Porto Alegre, Divisão de Neurologia, Porto Alegre RS, Brazil.; 6 Hospital da Restauração, Divisão de Neurologia, Recife PE, Brazil.; 7 Universidade Federal de São Paulo, Escola Paulista de Medicina, Departamento de Neurologia e Neurocirurgia, São Paulo SP, Brazil.

**Keywords:** Brain, Mental Health, Cognition, Primary Prevention, Environmental Hazards

## Abstract

Brain health is an emerging concept that is gaining international attention. It encompasses cognitive, sensory, social-emotional, behavioral, and motor functions that enable people to reach their maximum potential throughout all stages of life. With substantial preventable components, neurological diseases—including stroke and dementia—are the second largest cause of death worldwide and of disability-adjusted life years (DALYs). Studies continue to underscore the impact of environmental factors, social connections, lifestyle choices, and cardiovascular health on brain health outcomes. Major programs like the Intersectoral Global Action Plan (IGAP) on epilepsy and other neurological disorders of the World Health Organization (WHO), the American Heart Association's Life's Simple 7 (now Life's Essential 8), and national policies in Uruguay, Switzerland, and Norway emphasize the importance of multidisciplinary care and early prevention. At the community level, programs such as the Finnish Geriatric Intervention Study to Prevent Cognitive Impairment and Disability (FINGER) and its global variants emphasize the value of multifactorial interventions suited to the prevalence of social determinants among each population. It is crucial to encourage the incorporation of brain health into professional training, policy development, and public health frameworks. Despite improvements in stroke prevention, Brazil still has gaps in the research and promotion of brain health. To meet the global objectives for brain health, it is imperative to consistently invest in interdisciplinary research, public education, and equitable access to care in order to promote healthier aging and a better quality of life. The present review examines the various aspects of brain health determinants and offers neurologists and other medical professionals the most recent information by emphasizing prevention strategies, early childhood care, and holistic approaches that go beyond disease management.

## INTRODUCTION

Brain health is an evolving concept and a topic of global debate. The current definition proposed by the World Health Organization (WHO) is as follows: “the state of brain functioning across cognitive, sensory, social-emotional, behavioral, and motor domains, allowing a person to realize their full potential over the life course, irrespective of the presence or absence of disorders”. 1 The term first appeared in the literature in 1989, preceding the designation of the “Decade of the Brain” in the United States from 1990 to 1999. 2 However, brain health only became exponentially popular in the last years, 1 as the coronavirus disease 2019 (COVID-19) pandemic raised awareness of the link involving mental, social, and brain health. 3 When considering brain health, the goal is to bring an opportunity to achieve the brain's full potential as the conductor of the human body, not just to prevent neurological diseases.

From a collective perspective, the relevance of neurological diseases is undeniable. With stroke and dementia accounting for almost 8 million deaths worldwide, neurological diseases rank second in terms of causes of death and disability-adjusted life years (DALYs). Additionally, 80% of strokes and 45% of dementia cases can be attributed to preventable factors. 1 4 5 6 7 8 9 Demographic changes, such as population aging, and the significant financial costs involved, increase the urgency of these interventions. In Brazil, the annual direct and indirect costs of dementia are of US$16,548.24 per patient. 10 Regarding stroke, public spending averages USD 120 million annually, primarily driven by hospital expenses. 11 By 2040, it is expected that 50% of DALYs will be linked to neurological diseases. In lower-income countries, the burden is correspondingly higher. 12

From an individual perspective, there is a growing interest in achieving healthy aging and a better quality of life. 13 People are increasingly concerned with caring for their own and their families' health, recognizing that this care can significantly impact the physical health and emotional well-being of everyone involved. 14

There is a progressive rise in the burden of neurological diseases, and the need to maintain the function of the body's primary organ becomes more urgent, as people with brain diseases may experience death 20 years earlier than those without dysfunctions. For instance, 1 in 10 people older than 65 years of age suffers from dementia, one of the most debilitating illnesses in life, 9 and more than 85% of the 55 million individuals with dementia do not receive any postdiagnosis care. 15

Currently, healthcare providers' training emphasizes illness treatment instead of health promotion. However, as this approach is frequently insufficient to address all health needs, these professionals must adopt an alternative perspective and improve their understanding of how individuals can preserve and improve their brain health. Consequently, regardless of the existence of a disease, it may enable the patients to better realize their full potential. Considering neurologists as specialists in nervous system care, brain health must be part of its training and practice. A more equitable distribution of professionals across all Brazilian regions could enhance brain health, given the recent surge in the number of practitioners. 16

Articles indexed in PubMed containing the keyword *brain health* were selected, beginning from the inception of the concept in 1990; among these, the most pertinent articles from the past 10 years were prioritized for inclusion. The objective of the present review is to provide the neurologist and other healthcare professionals with the current knowledge on brain health.

## WHAT IT IS – UNDERSTANDING BRAIN HEALTH

In the 1990s, the United States Congress designated that period as the “Decade of the Brain” to foster awareness about the benefits of brain health research. This initiative resulted in the exponential growth of the Society for Neuroscience, with approximately 1 thousand new members annually, encouraging cooperation among researchers, scientists, patient advocacy organizations, and policymakers. 1 17 Over time, the notion of brain health has evolved, moving beyond the mere absence of illness. The WHO broadened the concept in 2022 to encompass the brain's capacity to form strong neural connections and function in socioemotional domains, highlighting the significance of exploiting prevention opportunities within the social determinants of health ( Table 1 ). Moreover, Hachinski 3 underscores the importance of safe, healthy, and supportive environments for development, and draws attention to the substantial financial benefits of brain health prevention.

**Table 1:** World Health Organization (WHO)Pillars to Achieve Mental Health 1

Physical health	Maternal health, intrauterine environment, genetic factors, nutrition, infections, noncommunicable diseases, health behaviors, and traumatic injuries.
Healthy environments	Safe use of chemicals and radiation, healthy and safe workplaces and agricultural practices, air and water quality, stable climate, and access to preserved nature and health-supportive built environments.
Safety and security	Physical safety and financial security during humanitarian crises and emergencies.
Learning and social connection	Education, lifelong learning, nurturing care, social connection to avoid social isolation, and social networks.
Access to quality services	Integrated care at all health/social care levels, skilled workforce and interdisciplinary teams, access to essential medicines, diagnostics and health products, and carer support.

In line with the American Heart Association's (AHA) Life's Simple 7 (LS7) tool to evaluate cardiovascular health (CVH), 18 Gorelick et al. 14 defined brain health as the capacity to adapt functionally to the environment. This definition is based on a longitudinal and encompassing approach that is assessed using individual capabilities and cognitive domains. The Centers for Disease Control and Prevention (CDC) and the Alzheimer's Association 19 related the term to reaching the full potential of the brain, although with a particular emphasis on the cognitive domain.

Brain health encompasses various aspects, with current definitions tending to suggest an approach that goes beyond the cognitive and structural functioning of the organ. This more holistic approach to health emphasizes longitudinal prevention starting early in life; an ideal state of cognitive, mental, and social functions supported by a safe and healthy environment. 1 3 In contrast, mental health, a field that may share similarities with brain health, places greater emphasis on the emotional and functional components of well-being, “a state that enables people to cope with the stresses of life, realize their abilities, learn well and work well, and contribute to their community”. 20

## HOW IT IS – DETERMINANTS OF BRAIN HEALTH

**Figure 1:**
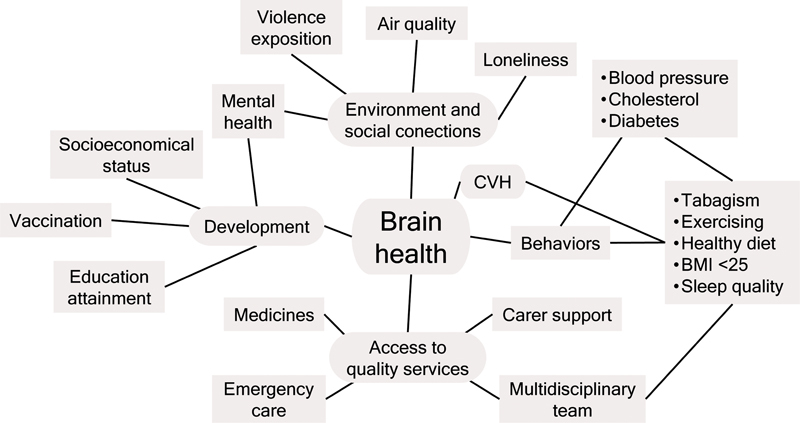
Abbreviations: CVH, cardiovascular health; BMI, body mass index. Determinants of brain health.

Countless factors affect brain health; some are personal, while others are environmental and beyond the individual's control ( Figure 1 ). Most of the information in the current review will pertain to variables that the patient and the healthcare provider can monitor and change, with a special focus on prevention.

Prior to introducing more specific strategies, it is crucial to understand that traditional cardiovascular risk factors have a direct impact on brain health. This was evident in 2010's AHA LS7. 18 This initiative used seven risk factors that can be changed through lifestyle choices to define ideal CVH. Aiming to reduce cardiovascular events and strokes by 20% in the American population within 10 years, LS7 includes 3 health factors (blood pressure < 120/80 mmHg, total cholesterol < 200 mg/dL, and fasting glucose < 100 mg/dL) and 4 behaviors (not smoking, exercising, keeping a body mass index [BMI] < 25 kg/m ^2^ , and following a healthy diet). Though the 10-year goal was not entirely achieved in 2020, a considerable 15.1% reduction in age-adjusted cardiovascular disease (CVD) mortality was observed. 21 Research 22 has stated that individuals with a poor LS7 score have a lifetime CVD risk of 43.1%, compared to 16.6% for those with an ideal score.

Recent research supports the importance of these CVH factors, showing their critical role in the prevention of neurological disorders such as dementia, stroke, migraine, and multiple sclerosis (MS). A meta-analysis 23 of 287,058 participants assessed AHA's LS7 CVH metrics relative to the cognitive aspect. Overall, a 6% decline in incident dementia was observed for every point increase in CVH score. Besides that, there was a J-shaped dose-response curve in the relationship between late-life CVH score and dementia risk. This implies that following the AHA's ideal CVH recommendations may not only protect against cardiovascular disease, but also against cognitive aging and dementia. 23

Furthermore, in 2022, the American Heart Association (AHA) issued a presidential advisory that included sleep health in the LS7, which is now known as “Life's Essential 8 (LE8)”. 24 A score based on this new approach to CVH was used in 2023 25 to analyze 2 independent Chinese cohorts for the incidence of strokes in the future. Among 68,854 participants, the risk of stroke was 67% lower for those with an ideal LE8 CVH score (≥ 80 points) than for those with a poor score (< 50 points) (hazard ratio: 0.33 [95%CI: 0.20–0.54]).

Recent research 26 has demonstrated that these variables can also affect the prevalence of migraine, the second most common cause of disability, impacting 1.04 billion people worldwide. In total, 332,895 people from a United Kingdom (UK) biobank had their migraine risk assessed over a median follow-up of 13.5 years. According to the cohort, adhering to a higher category of LE8 has been found to lower the migraine risk by 22%. Among the individual lifestyle factors, sleep duration and patterns had the greatest impact, lowering the risk of migraine by 10.16% and 16.39% respectively.

Chronic inflammation and neuronal loss are hallmarks of MS, the world's leading cause of non-traumatic disability in young and middle-aged adults. Even though the underlying immunopathologic processes are well understood, extensive research 27 has disclosed that cardiovascular risk factors may contribute to the development and progression of this disease. Additionally, physical inactivity, obesity, cigarette smoking, low sunlight exposure/low serum vitamin D levels, and Epstein-Barr virus infection are behavioral risk factors currently known to have a causal role in MS development. Furthermore, diabetes, dyslipidemia, hypertension, and obesity are associated with the disease's progressive forms.

As single risk factors, high blood pressure, obesity, diabetes, smoking and alcohol are known to increase events of stroke and dementia. Systemic arterial hypertension accounts for 60% of stroke-related DALYs worldwide, 7 high BMI is associated with more than 20%, 26 and high insulin resistance (assessed through high fasting sugar) is associated with more than 10%. Smoking, which affects 9.3% of the Brazilian population, is globally responsible for 21% of stroke-related DALYs, and it has been connected to various neurological conditions, including dementia and the exacerbation of MS. 28 29 Meanwhile, alcohol abuse affects 20.8% of the Brazilian population, and it is linked to more than 200 diseases, encompassing 3 million deaths annually. 9 29

On the other hand, positive behaviors could preserve the integrity of brain health. Even patients with chronic neurological diseases such as MS could improve some cognitive abilities with simple life changes such as household tasks. 30 About 2% of dementia cases (5% in Latin America) and 8% of stroke-related DALYs are caused by sedentary behavior. Exercise promotes astrocytic proliferation and mitochondrial function, decreases autophagy, and increases the brain-derived neurotrophic factor (BDNF), increasing neuroplasticity. 31 32 Additionally, exercise increases insulin-like growth factor 1 (IGF-1), which enhances intraneuronal metabolism and neuronal insulin sensitivity. Adults who engage in physical activity are 35% less likely to experience cognitive decline. 14 The Global Council on Brain Health (GCBH) 33 recommends 150 minutes of moderate-intensity aerobic exercise and at least 2 days of moderate strength training per week, a goal achieved only by 40.6% of the Brazilian population. 29

Dementia and cognitive decline have been linked to chronic sleep deprivation. Fragmented sleep is associated with a higher risk of small-vessel stroke and worse emotional and cognitive functioning in older adults. 34 Between the 6th and 7th decades of life, fewer than 6 hours of sleep can lead to a 30% increased risk of dementia. 31 Sleep affects brain health through several mechanisms, such as improving the clearance of toxic proteins (phosphorylated tau and beta-amyloid), mostly through the glymphatic system, and reducing synaptic activity and metabolic rate. 31

General vascular risk factors (dyslipidemia, diabetes, and hypertension) enhance the production and decrease the clearance of beta-amyloid and tau proteins, which are associated with damage to the neurovascular unit (NVU). The presence of hemoglobin and iron, which are linked to oxidative stress and inflammation, results in neural damage when the blood-brain barrier (part of the NVU) breaks down. These factors may be associated to the early pathophysiology of Alzheimer's disease (AD) and pose as early indicators of executive dysfunction. 35

A lower risk of AD, CVD, and type-2 diabetes are linked to specific diets, including the Dietary Approaches to Stop Hypertension (DASH, characterized by low sodium intake), the Mediterranean diet (rich in monounsaturated fats, grains, and fish), and the Mediterranean-DASH Intervention for Neurodegenerative Delay (MIND) diet. 34 Conversely, a “Western diet” consisting of processed, high-fat foods is directly correlated with depression and cognitive decline. Damage from a high-fat diet is believed to be related to mitochondrial dysfunction in the hippocampus. Additionally, dyshomeostasis of cholesterol might aggravate neuroinflammation, a known factor in the pathophysiology of AD. 36 In a UK biobank prospective cohort, 37 increased intake of ultraprocessed food was associated with a 14% higher risk of AD, 28% higher risk of vascular dementia and 25% higher risk of dementia.

A global debate surrounds the relationship between vitamins and dementia. Vitamin D deficiency has been linked to a 1.42-fold increased risk of dementia and a 1.57-fold increased risk of AD. 38 In patients with mild cognitive impairment, complex B vitamins have been found to be generally beneficial for overall cognitive function, with the greatest benefits occurring during the first few months of treatment. On the other hand, no obvious immediate advantages were found for antioxidant vitamins. 39

Environmental factors additionally play an important role, as 99% of the world's population is exposed to air pollution. Poor air quality is considered to raise the risk of hemorrhagic stroke by up to 31% in patients with chronic illnesses. 40 Besides this, during pregnancy and early childhood, exposure to pollution reduces gray matter volume and enlarges the amygdala. 41 An additional environmental issue is the growing concern about microplastic pollution and its presence in human tissues. These polymers, which can originate from paint, cosmetics, and synthetic fibers, have been identified in the olfactory bulb of the human brain and have raised concerns regarding potential neurotoxicity. 42

Endothelial diseases may be linked to social and environmental factors, but these effects can be reversed, offering chances to enhance brain health. 6 Given that all dementias exhibit some degree of vascular causation, particularly as a result of endothelial damage, the intersection of vascular factors and dementia presents new therapeutic opportunities. 35 The ability to adapt and continue to function in the face of damage is becoming a more important part of the concept of brain health. Neuronal connectivity can be promoted, and brain resilience can be improved through adult socialization and cognitive training. 7 31

Among non-traditional risk factors, social connections have recently been found to have a direct effect on brain disease. The feeling of loneliness (the gap between desired and actual social contact) increases the risk of cognitive decline. 34 For diabetic individuals, environments with higher divorce rates have increased the risk of hemorrhagic stroke by 3.9 times. 40 Overall, interpersonal relationships are associated with greater brain volume. Having a purpose in life reduces the risk of dementia by 20%, 34 while social isolation increases the risk of cognitive impairment. 43 Having a pet has been demonstrated to decrease verbal aggression and anxiety in AD patients, illustrating the profound effects that even small changes can have on brain and mental health. 34

Regarding dementia, population studies 44 also show that common vaccines, such as the ones against herpes zoster and tetanus, diphtheria, and pertussis (Tdap), are linked to a decreased risk of dementia. Additionally, a meta-analysis 45 has found that other adult vaccinations (against influenza, hepatitis A and B, and typhoid) are linked to a notable decrease in dementia risk, supporting the notion that vaccination may be a helpful method to prevent dementia.

The Norwegian experience 46 from 1990 to 2019 illustrates the combined significance of these determinants: behavioral risk reductions resulted in a 5.4% decrease in dementia, a 30% decrease in myocardial infarction, and a 35% decrease in the incidence of stroke.

A Brazilian study 47 examined the impact of a comprehensive program called “Oficina da Lembrança” (“Memory Workshop”), which involves social interaction, physical activity, digital inclusion, and cognitive stimulation. Although there were no discernible changes in the biomarkers of neurodegeneration, the results indicated that participants in the intervention group had significantly improved cognitive function.

A mnemonic device, “SAFEST BRAINS,” was recently proposed to highlight key factors for neurologists to assess in daily practice regarding brain health. “SAFEST” corresponds to Sleep, Affect (mental health), Food (nutrition), Exercise, Social interaction, and Trauma prevention. “BRAINS” emphasizes Blood pressure control, metabolic/genetic Risks, Affordability and Adherence, Infection prevention, minimizing Negative exposures, and addressing Social and structural determinants of health. 48

## WHEN TO START IT – TIME FOR BRAIN HEALTH

**Figure 2:**
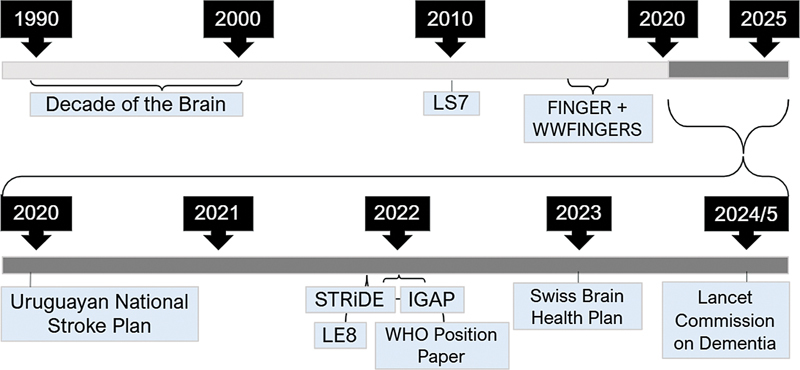
Timeline of advancements in brain health.

Brain health concepts progressively emphasize care starting prior to conception and throughout development, maturation and aging, underscoring the importance of the environment to which the individual is exposed ( Figure 2 ). According to research on humans and animals, the brain is more susceptible to stress in the early stages of life. These difficulties can affect the corticostriatal circuit and lead to persistent deficits in reward processing, which can have detrimental effects later in life. 49 50

Poverty, discrimination, and marginalization are significant well-known factors that raise the risk to brain health by influencing gene expression and neural signaling throughout life. 50 As demonstrated in adolescents exposed to neighborhood violence, 50 verbal abuse during development has been related to emotional dysregulation and hippocampus atrophy. 6 Lower parental socioeconomic status is associated with lower levels of education and cognition in adulthood, while low socioeconomic status raises the risk of dementia, epilepsy, and infections in the nervous system. 6

The lack of support in adverse circumstances can increase the amygdala's activity. Furthermore, long-term stress in adults can weaken cortical structures by hyperactivating the hypothalamic-pituitary-adrenal axis. 50 While lower amygdala activity is linked to a lower risk of cardiovascular events, elevated amygdala activity triggered by stress is linked to fat accumulation and diabetes. 51

In their 2024 update, 52 the Lancet Commission on dementia used population databases and meta-analyses to estimate the proportion of dementia cases that could be avoided if the risk factors under investigation were eradicated (population attributable fraction, PAF). They found that 5% of dementia cases could be avoided by reducing low educational attainment in childhood. Hearing loss is the most common risk factor in midlife, making up 7% of the total, followed by a 3% reduction for depression, traumatic brain injury, and elevated low-density lipoprotein (LDL) cholesterol. In later life, social isolation accounts for 5% of the PAF, making it the main determinant, followed by air pollution at 3% and vision loss at 2%. The study 52 suggests that 45% of dementia cases might be prevented by lowering these factors. The estimated overall PAF for dementia in the Brazilian population is of 48.2%. The highest PAF (7.7%) is related to lower educational attainment, followed by hearing loss (6.8%) and hypertension (7.6%). 53 These results confirm the importance of brain care during all periods of life.

Care ought to be integral and longitudinal. The brain develops rapidly early in life and keeps adapting throughout the lifespan. The greatest potential for neuroplasticity takes place in early childhood. Over time, health determinants affect how the brain reacts to stress and adversity. Starting to optimize brain health care since before conception enables individuals to achieve their full potential for development and quality of life. 1

## HOW IT IS BEING DONE – CURRENT STUDIES ON BRAIN HEALTH

Over the past 10 years, there has been a notable increase in interest in brain health, leading to important discoveries about the pathophysiological mechanisms of dementia syndromes. Cognitive medicine research aims to comprehend the molecular and cellular processes that underlie cognitive function. Recent research 5 31 has emphasized the proven importance of the NVU, particularly in the brain's ability to heal and manage injuries to avoid deficits. Conversely, it has been found that the glymphatic system is largely responsible for removing waste from the brain, especially while sleeping. The mechanism facilitates the passage of cerebrospinal fluid through perivascular spaces, where it combines with interstitial fluid to rid the brain parenchyma of metabolic waste, including beta-amyloid. These processes impact immune surveillance and growth-factor synthesis, maintaining the brain's microenvironmental homeostasis. 14 31 54

Government initiatives for brain health are already being implemented in several developed countries. Based on established risk and protective factors, a new national escalation plan for mental health in Norway was introduced for 2023 to 2033: 55 its main objectives are universal mental health, prenatal screening, training for all new daycare center teachers, and mental health as a school subject. Between 2002 and 2013, the incidence of dementia in Ontario decreased by 7.4% because of the implementation of primary risk prevention strategies for cerebrovascular diseases. 56

In 2023, the Swiss Brain Health Plan (SBHP) 2023 to 2033 57 was introduced, offering a more comprehensive strategy to prevent a range of brain disorders. By planning public policies, training programs for healthcare providers, and scientific events on specific topics (such as dementia, sleep, stroke, and psychiatry) across the country, the initiative seeks to promote brain health across all stages of life. Research on the impact of brain health determinants in the Swiss population is also encouraged by the SBHP, which seeks to create an individualized, person-centered and cost-effective public health system. 57

Besides this, some strategies still focus mainly on systemic diseases, not considering brain health. By 2050, the prevalence of dementia is expected to increase by 116% in wealthy nations and by 264% in underdeveloped ones. 13 Nearly 2 million people in Brazil suffer from dementia now, and the average monthly direct and indirect costs per person for the moderate stages of dementia are estimated to be of R$6,700. 10 Access to a multidisciplinary health team and caregiver support are essential, since up to 50% of caregivers experience depression. In 2022, the WHO 1 launched iSupport, a program that aims to reduce mental and physical health problems among caregivers by offering educational modules on daily care and managing behavioral changes.

In low- and middle-income countries (LMICs), the Strengthening Responses to Dementia in Developing Countries (STRiDE) program seeks to enhance dementia care, treatment and support networks. Brazil has joined this initiative, which aims to improve the lives of those with dementia while preventing family members and other caregivers from incurring excessive expenses or compromising their own health. With the core STRiDE team in Brazil based at Universidade Federal de São Paulo (UNIFESP) and with support from the Brazilian Federation of Alzheimer's Disease Associations (Federação Brasileira das Associações de Alzheimer, FEBRAZ, in Portuguese), the program aims to foster research focusing on gaps in care provision for progressive cognitive impairment. 58 59

In Brazil, the National Policy to Address Alzheimer's Disease and Other Dementias was enacted through Law no. 14,878/2024. 60 In addition to strengthening primary health care and improving education for professionals involved in patient care, the policy's guidelines also seek to raise awareness and promote healthy lifestyle choices. The program is founded on comprehensiveness and interdisciplinarity and complies to international guidelines, particularly the Global Action Plan on the Public Health Response to Dementia 2017–2025. Additionally, it encourages the integration of existing programs and services to create a decentralized, efficient care pathway for dementias. 60 Over the last 20 years, the Brazilian Academy of Neurology (Academia Brasileira de Neurologia, ABN, in Portuguese) has actively participated in public awareness campaigns, scientific events, and political discussions regarding brain-related diseases and their impact on the community.

Davos Alzheimer's Collaborative 61 (DAC) is an additional organization that supports dementia care and research. Under the leadership of the Global Chief Executive Officer Initiative on Alzheimer's Disease (CEOi) and the World Economic Forum, DAC presents a clinical trial-ready system to develop therapies for the prevention and treatment of cognitive impairment. The organization's goal is to create an international cohort system, which will connect trial sites globally, to increase knowledge on Alzheimer's among various populations. 58 59

In March 2020, Uruguay developed a National Stroke Plan. 62 Its activities, which include immunizations, new guidelines, and the encouragement of healthy behaviors, concentrate mainly on prevention and education from an early age. Additionally, multidisciplinary teams with expertise in vascular events were assembled into 42 Stroke Ready Centers. The strategy, which aims to provide universal access to stroke prevention, treatment, and rehabilitation, has already demonstrated improvements in access to recanalization therapies. 62

Despite the absence of a brain health plan in the Brazilian context, significant progress is being made. The National Stroke Project and the National Stroke Law of 2012 have increased the number of stroke centers nationwide, providing funds for acute care and rehabilitation. 63 It has also offered training for healthcare providers and launched campaigns to increase public awareness of strokes.

Considering preventive strategies based on lifestyle, the Finnish Geriatric Intervention Study to Prevent Cognitive Impairment and Disability 64 65 (FINGER) showed that multifaceted personalized interventions, including cognitive training, maintaining and increasing cardiovascular fitness, lowering cardiovascular risk factors, and adopting a healthy diet, were successful in preventing cardiovascular disease and enhancing general health. This Finnish study compared general health recommendations with these high-intensity structured interventions, and it found improvements in executive function and processing speed in the intervention group, while also proving to be cost-effective in a healthcare model.

Despite the promising outcomes of the FINGER trial, other multidomain randomized trials, such as the Dutch Prevention of Dementia by Intensive Vascular Care (PreDIVA) and the French Multidomain Alzheimer Preventive Trial (MAPT), failed to demonstrate any apparent benefits. 7 In 2017, the World-Wide FINGERS (WW-FINGERS) project 66 was launched to further study these interventions. This network aims to optimize and adapt the FINGER multidomain lifestyle model to different environments and assess the viability and efficacy of FINGER-based protocols across a range of environments and populations.

Twelve Latin American countries, including Brazil, are taking part in the LatAm FINGER initiative. 67 A sample of 100 patients from each nation will be included in the study, which is currently in the recruitment phase. The study will examine the effects of interventions (cognitive and physical training, the MIND diet, socialization, and regular medical examinations) on a global cognition composite (the Latin American Neuropsychological Test Battery) in individuals who are at high risk of cognitive decline due to a sedentary lifestyle and metabolic-cardiovascular profile. 66 67

These multifaceted interventions are better suited for each person's unique preventive potential than general recommendations. However, since it is not yet feasible to implement personalized prevention for entire populations (particularly in low-income countries), health promotion should first focus on social and political changes that increase awareness of risk factors. 7

## HOW CAN IT BE ACHIEVED – BRAIN HEALTH POTENTIAL

Objective measurements of brain health are essential to enhance prevention and treatment. To this end, “The Braincare Score” 68 was developed to assist in establishing practical parameters on personal and global levels. The score is already a useful tool for future research and initiatives, since it has been demonstrated to predict depression, dementia, and stroke even in cases in which there are high genetic risks. 69 70

The CDC and the Alzheimer's Association 71 partnered to create a roadmap to advance cognitive health. Though the document's primary focus is on maintaining cognitive abilities, it also outlines 25 actions that can improve brain health when implemented across four public health domains: education/population empowerment, policy/partnership development, ensuring a competent workforce, and monitoring/evaluation.

The WHO introduced the Intersectoral Global Action Plan (IGAP) on epilepsy and other neurological disorders for 2022 to 2031. 4 The strategy's core objective is to promote prompt and efficient diagnosis and treatment for these conditions. By 2031, it is expected that 75% of nations will have integrated neurological disorders into their universal healthcare system, and 80% will offer basic care with the essential medicines and technologies.

The IGAP's guiding principles are universality, continuity of care, empowerment and the participation of individuals with neurological disorders in the development of public policies. To evaluate the success of the measures, the intended objectives are to double global research on neurological diseases by 2031 and ensure that key indicators of neurological diseases are collected every 3 years in 80% of countries. 4

However, most nations are far from reaching these goals. In this sense, the OneNeurology Partnership 12 was formed to prioritize IGAP's 4 objectives. Improving governance should be the focus, especially in low-income countries, since less than 30% of them have any laws on neurological conditions. By 2031, 75% of nations are expected to have updated their national policies or strategies to include neurological diseases, and 100% are expected to have conducted at least 1 program or awareness campaign about these conditions.

Winter et al. 12 proposed six strategic drivers to be adopted by the IGAP member states to achieve the endpoint. Among these, the emphasis on exploiting strategic entry points in cases of neurological diseases to link patients to brain health services stands out, as well as multisector partnerships, known as the six Ps coalition: patients, healthcare service and product providers, policymakers, payors, implementation partners, and the general population.

The American Academy of Neurology's (AAN) Call to Action 72 explains and details the importance of research, education, and direct-to-public messaging throughout the lifespan of an individual. It also promotes the “AAN Brain Health Platform,” with emphasis on increasing public engagement, optimizing brain health by implementing preventative measures, and accelerating scientific research through interdisciplinary collaborations.

For brain health to become more achievable globally, guidance can be drawn from the pillars proposed by the WHO ( Table 1 ). 1 These are in line with other plans and include shared components such person-centered support, public and health professional education, and caregiver support. These strategies involve global interdisciplinary approaches, focusing on research in basic fields (neurophysiology, brain function, and injury mechanisms) and applied research (health promotion strategies, brain resilience mechanisms, risk identification, and evidence-based interventions).

Long-term research and initiatives such as the WW-FINGERS and STRiDE should be supported from a public health standpoint to identify the health factors and the most effective strategies for each population. Such efforts enable the suggestion of feasible approaches to meet the WHO's IGAP, especially in low-income nations, where the burden of these illnesses is much more pronounced. 4 58 59 66

In conclusion, it is becoming clear that prevention is the best course of action in all areas of health, and brain care should be no different. In both public health and routine clinical practice, prevention, treatment, and rehabilitation are crucial.

Shifting the prevailing mindset and clinical approach among physicians engaged in this field, whether in academic settings or among specialized practitioners, will represent a significant challenge. Vigilance should remain continuous throughout life, considering the growing evidence of the impact of an individual's environment on brain development. This is particularly essential in the early stages, when the brain exhibits greater neuroplasticity.

Considering the significant financial, psychological, and physical expenses linked to neurological illnesses, governments need to give global planning projects top priority. Despite Brazil's progress in stroke prevention along with assisting those suffering from dementia, the nation is still lagging in brain health research and the WHO's goals for neurological illness control. This calls for incorporating public policies concerning awareness of these diseases into national health legislation and guidelines. Additionally, a competent multidisciplinary team must perform a successful and innovative approach to person-centered disease control and health determinant surveillance in all age groups.
